# Perceived stress predicts altered reward and loss feedback processing in medial prefrontal cortex

**DOI:** 10.3389/fnhum.2013.00180

**Published:** 2013-05-20

**Authors:** Michael T. Treadway, Joshua W. Buckholtz, David H. Zald

**Affiliations:** ^1^Department of Psychiatry, Center for Depression, Anxiety and Stress Research, McLean Hospital/Harvard Medical SchoolBelmont, MA, USA; ^2^Department of Psychology, Harvard UniversityCambridge, MA, USA; ^3^Departments of Psychology and Psychiatry, Vanderbilt UniversityNashville, TN, USA

**Keywords:** medial prefrontal cortex (mPFC), perceived stress, reward processing, insula, Monetary Incentive Delay task

## Abstract

Stress is a significant risk factor for the development of psychopathology, particularly symptoms related to reward processing. Importantly, individuals display marked variation in how they perceive and cope with stressful events, and such differences are strongly linked to risk for developing psychiatric symptoms following stress exposure. However, many questions remain regarding the neural architecture that underlies inter-subject variability in perceptions of stressors. Using functional magnetic resonance imaging (fMRI) during a Monetary Incentive Delay (MID) paradigm, we examined the effects of self-reported perceived stress levels on neural activity during reward anticipation and feedback in a sample of healthy individuals. We found that subjects reporting more uncontrollable and overwhelming stressors displayed blunted neural responses in medial prefrontal cortex (mPFC) following feedback related to monetary gains as well monetary losses. This is consistent with preclinical models that implicate the mPFC as a key site of vulnerability to the noxious effects of uncontrollable stressors. Our data help translate these findings to humans, and elucidate some of the neural mechanisms that may underlie stress-linked risk for developing reward-related psychiatric symptoms.

## Introduction

Alterations in reward-seeking and goal-directed behavior are a common symptom of mental illness. In the broadest sense, such alterations usually reflect a shift in how different options in the environment are valued and pursued, resulting in either a reduced motivation for experiences that were previously found to be rewarding (Treadway and Zald, in press), or a heighted sense of craving for particular rewards (e.g., drugs, food) (Volkow, 2004; Everitt and Robbins, 2005). While progress has been made in identifying the neural systems that participate in reward processing behavior, many questions remain as to how these systems become dysfunctional in clinical populations.

Exposure to stress is a central risk factor in the development of psychiatric conditions characterized by prominent abnormalities in reward-related processes, such as depression, schizophrenia, and substance use (Kessler, 1997; Kendler et al., 1999, 2004; Sinha, 2001, 2008; Yuii et al., 2007). The term stress describes physically or emotionally demanding circumstances, frequently involving the real or imagined threat of loss or pain (McEwen, 2007). This can include either physical or emotional pain, and may occur in the context of professional, social and familial relationships. A wealth of data suggests that stress exposure alters how individuals process and make decisions about rewards in their environment (Bogdan and Pizzagalli, 2006; Koob and Kreek, 2007; Pascucci et al., 2007; Pizzagalli et al., 2007; Arnsten, 2009; Dias-Ferreira et al., 2009; Schwabe and Wolf, 2009; Cavanagh et al., 2010; Cabib and Puglisi-Allegra, 2011; Mather and Lighthall, 2012; Shafiei et al., 2012). In particular, stress has been found to blunt sensitivity to new information about future rewards, a phenomenon that has been demonstrated across a variety of experimental paradigms. For example, under conditions of elevated stress, subjects were less sensitive to reinforcement contingencies during a signal-detection paradigm (Bogdan and Pizzagalli, 2006; Pizzagalli et al., 2007; Bogdan et al., 2011). Similarly, subjects show diminished reinforcer devaluation immediately following stress, suggesting that stress can produce habitual response patterns that are resistant to changes in external or internal conditions (e.g., satiety) (Dias-Ferreira et al., 2009; Schwabe and Wolf, 2009; Lemmens et al., 2011).

A variety of evidence highlights a corticostriatal circuit encompassing the striatum and medial prefrontal cortex (mPFC) as a critical neurobiological substrate for stress-borne alterations in reward processing. Data from preclinical studies suggest that stress produces rapid changes in catecholamine levels (Abercrombie et al., 1989; Pascucci et al., 2007), gene expression (Ons et al., 2010; Wang et al., 2010), and local circuit remodeling (Arnsten, 2009) within these areas. Corroborating observations have been made in human neuroimaging studies; where stress has been shown to increase dopamine release (Scott et al., 2006; Soliman et al., 2008; Lataster et al., 2011) and alter neural responses to reward decision-making and anticipation (Ossewaarde et al., 2011; Mather and Lighthall, 2012; Schwabe et al., 2012).

While these studies have helped identify the systems-level mechanisms that underlie responses to an acute stressor, they generally do not address questions regarding the biological basis of individual differences in how stressors are perceived. This issue is critical for understanding how stress confers risk for developing psychopathology, as epidemiological studies reveal that individuals who consider stressful experiences to be uncontrollable and overwhelming are substantially more likely to develop psychiatric symptoms following stress exposure (Kendler et al., 1993, 2004; Kessler, 1997). This is particularly true for symptoms related to impaired reward-reward processing, such as anhedonic symptoms in depression and schizophrenia (Kuiper et al., 1986; Docherty, 1996; Myin-Germeys et al., 2001; Hammen, 2005; Myin-Germeys et al., 2005; Phillips et al., 2005; Rao et al., 2009). Highlighting the importance of this distinction, rodent models suggest that uncontrollable stressors produce a unique pattern of neurobiological changes, particularly in the mPFC (Cabib and Puglisi-Allegra, 1994, 2011; Bland et al., 2003; Amat et al., 2005; Maier and Watkins, 2010). As compared to controllable stressors (i.e., paradigms where instrumental action may alleviate the stressor), repeated exposure to uncontrollable stressors can result in learned helplessness behavior and anhedonia (Seligman et al., 1968; Willner et al., 1992a,b; Amat et al., 2008).

The effects of recent stress perceptions on reward-processing in otherwise non-stressful contexts has not been well-characterized. Recent neuroimaging work in humans has focused on the use of experimental paradigms that combine measures of reward processing with laboratory stress manipulations, which can elucidate some of the neural mechanisms underlying changes in reward-related behavior immediately following exposure to stressful stimuli (Ossewaarde et al., 2011; Mather and Lighthall, 2012; Porcelli et al., 2012). However, fewer studies have examined how such networks are affected by perceptions of stress over a longer time period. Consequently, the goal of the current study was to explore associations between reward processing and reported perceptions of stressors in the preceding month. The advantage of this design is its ability to explore the consequences of recent levels in perceived stress on neural networks supporting reinforcement, which may help explain how prior stress exposure can alter reward circuitry so as to confer risk for the subsequent development of psychopathology.

To address this question, we recruited a sample of healthy community volunteers who completed a measure of perceived stress over the past month, and then performed a behavioral reward-processing task during a functional magnetic resonance imaging (fMRI) scan. Recent levels of perceived stress were assessed using the Perceived Stress Scale (PSS) (Cohen et al., 1983), a widely-used instrument that measures the frequency, severity, and perceived controllability of daily stressors over the previous 1-month period. The PSS has been previously linked to risk for the development of both physical and mental health symptoms (Kuiper et al., 1986; Cobb and Steptoe, 1996; Culhane et al., 2001), as well as elevations in stress hormones (Pruessner et al., 1999) and inflammation (Maes et al., 1999). More importantly for the aims of the current study, the PSS has been linked to alterations in reinforcement learning assessed using a signal detection task (Pizzagalli et al., 2007). To assess the effects of perceived stress on reward processing, subjects were scanned while performing a monetary-incentive delay (MID) task (Knutson et al., 2000). The MID is a well-validated neuroimaging paradigm that probes neural responses to anticipation of reward (i.e., motor preparation to pursue reward) as well as integration of reward feedback. While the former condition typically engages the ventral striatum, the latter condition recruits mPFC, including aspects of pregenual anterior cingulate cortex (ACC), anterior cingulate sulcus, and Broadmann area 10 (Knutson et al., 2003, 2005). Importantly, the MID has previously been used to identify alterations in neural responses to reward processing in psychiatric populations with reward-related symptoms (Juckel et al., 2006; Pizzagalli et al., 2009).

Given the evidence reviewed above that stress is associated with diminished sensitivity to reward information (Bogdan and Pizzagalli, 2006; Pizzagalli et al., 2007; Schwabe and Wolf, 2009; Bogdan et al., 2011) and that the striatum and mPFC may be particularly critical nodes involved in responses to perceived stress (Cabib and Puglisi-Allegra, 1994, 2011; Amat et al., 2005), the MID appears especially well-suited as a task to probe neural activity in reward-related networks that may be a priori predicted to be affected by levels of perceived stress.

## Methods

### Participants

Participants were 38 volunteers recruited from the community. Subject ages ranged from 18 to 34, with a mean age of 22. Roughly equal numbers of men (*n* = 20) and women (*n* = 18) participated. All subjects were screened for any contraindications for participation in an MRI study, e.g., obesity, claustrophobia, cochlear implant, metal fragments in eyes, cardiac pacemaker, neural stimulator, and metallic body inclusions or other metal implanted in the body, pregnancy.

### Measure of recent chronic stress

To assess recent levels of chronic stress, all subjects were administered the PSS. The PSS is a well-validated brief self-report measure that has been widely used as an index of current-levels of chronic daily-life stressors (Cohen et al., 1983). Subjects are asked to rate the frequency and intensity of stressful events that have occurred over the most recent one-month period. The PSS also incorporates items that ask subjects to rate the perceived predictability and controllability of these stressors, as well has how overwhelmed they felt. Examples of items from this measure include “In the last month, how often have you felt difficulties were piling up so high that you could not overcome them?” and “In the last month, how often have you felt nervous or ‘stressed?”’ Each item is rated using a 0–4 scale where 0 is defined as “never” and 4 is defined as “very often,” and scores are generated by summing across the total number of items (after appropriate reverse-coding for 4 of the 10 items). Internal reliabilities (Cronbach's-α) across the 10-item scale were recently reported to be 0.91 in two separate national surveys that each included 2000 participants (Cohen and Janicki-Deverts, 2012). The maximum score on this measure is 40, and the minimum is 0. While the PSS is not a clinical instrument and therefore has no “cut-off” score related to diagnostic categories, it has been found to predict mental and physical health outcomes, including vulnerability to infections disease (Cobb and Steptoe, 1996; Culhane et al., 2001) and depression (Kuiper et al., 1986). More specifically to the domain of reward processing, the PSS has been found to predict decreases in reward sensitivity using a signal-detection reinforcement task (Pizzagalli et al., 2007).

### Monetary incentive delay (MID) task

**Figure 1:**
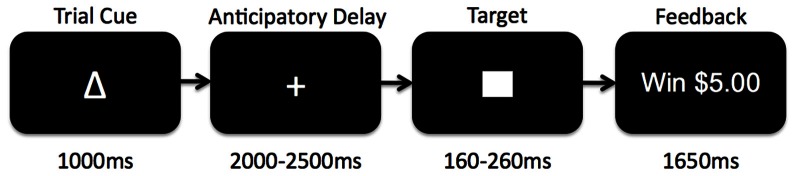
**Schematic diagram of the Monetary Incentive Delay (MID) task used in the current study.** Participants began each trial presented with 1 of 7 reward cues indicating whether they had an opportunity to gain reward, lose reward, or experience no change if they successfully pressed a button before a visual target disappeared on the screen. After the trial cue presentation, participants fixated on a centered cross-hair while waiting for the target to appear (anticipatory delay). The target would then appear for a variable amount of time during which subjects would attempt to press a button before the target disappeared. Afterwards, subjects received feedback as to whether or not they had been successful, and what the monetary outcome was for the trial.

The Monetary Incentive Delay (MID) task is a widely used assessment of neural circuitry associated with reward anticipation and processing of reward feedback (Knutson et al., 2000, 2003, 2005) (see Figure 1). Details of our MID task and fMRI scanning protocol have been published previously (Buckholtz et al., 2010). Briefly, during the task participants have the opportunity to win or lose money by pressing a button during the very brief presentation of visual target stimulus. For each trial, participants are shown one of seven cues, indicating that they have the potential to win money (reward magnitude range = $0.20, $1.00, $5.00; *n* = 74), the potential to avoid losing money (loss magnitude range = $0.20, $1.00, $5.00; *n* = 69), or that no money was at stake for that trial (No Change trials; *n* = 37). Subjects were instructed to fixate on a cross-hair during a variable interval of 2000–2500 ms (anticipatory delay phase), and then respond to a white target square that appeared for a variable length of time (target phase, 160–260 ms) with a button press. For Potential Win trials, participants were told that if they successfully pressed the button while the target was onscreen (a “hit”) they won the amount of money indicated by the cue, while there was no penalty for failing to press the button while the target was onscreen (a “miss”). For Potential Loss trials, participants were told that no money was won or lost for hits, but misses would lead to a loss of the amount indicated by the cue for that trial. A feedback screen (outcome phase, 1650 ms) followed the target's disappearance. The feedback screen notified participants how much money they won or lost during that trial, and indicated their cumulative total winnings at that point. Even though no money was at stake during the No Change trials, participants were instructed to rapidly press the button during the display of the target stimulus.

Before entering the scanner, participants completed a practice version of the task and were shown the money that they could earn by performing the task successfully. Based on reaction times obtained during the prescan practice session, target durations were adjusted such that each participant succeeded on approximately 66% of his or her responses. Each MID task session is comprised of 4 functional runs, each approximately 7.73 min long. The MID was programmed in E-Prime (http://www.pstnet.com/products/e-prime/) and run off of a dedicated Pentium computer from the scanner control room. The visual display was presented on an LCD panel and back-projected onto a screen positioned at the front of the magnet bore. Subjects lay supine in the scanner and viewed the display on a mirror positioned above them. Manual responses were recorded using a keypad (Rowland Institute of Science, Cambridge MA).

### fMRI data acquisition

All fMRI scans were performed on two identically configured 3 Tesla Phillips Achieva scanners located at the Vanderbilt University Institute for Imaging Science (VUIIS). T1-weighted high-resolution 3D anatomical scans were obtained for each participant (FOV 256 × 256, 1 × 1 × 1 mm resolution). Fast spin echo axial spin density weighted (*TE* = 19, *TR* = 5000, 3 mm thick) and T2-weighted (*TE* = 106, *TR* = 5000, 3 mm thick) slices were obtained to exclude any structural abnormalities. Additionally, a field map was additionally collected in order to remove distortion caused by inhomogeneity. Functional (T2^*^ weighted) images were acquired using a gradient-echo echoplanar imaging (EPI) pulse sequence with the following parameters: *TR* = 2000 ms, *TE* = 25 ms, flip angle 90°, FOV 240 × 240 mm, 128 × 128 matrix with 30 axial oblique slices (2.5 mm, 0.25 mm gap) oriented approximately 15 degrees from the AC-PC line. The slice prescription was adjusted for each subject to ensure coverage of the midbrain, ventral striatum, amygdala, mPFC, and orbital gyrus. Higher-order shimming was employed to compensate for magnetic field inhomogeneity in the orbitofrontal/ventral striatal region. fMRI volume acquisitions were time-locked to the offset of each cue and each target, so were thus acquired during anticipatory and during outcome periods. 242 volumes were acquired for each functional run.

### fMRI data preprocessing and analysis

Prior to random effects analysis in SPM5, all fMRI time series data received conventional preprocessing, including slice-timing correction, spatial realignment, normalization into a standard stereotactic space (MNI) and smoothed with a 6 mm full-width-half maximum gaussian kernel. Functional images were slice-time corrected using the middle slice as a reference, motion corrected via spatial realignment (4th degree B-spline) of all images to a mean image after alignment to the first image of each run. Following realignment, the Fieldmap toolbox was used to create voxel displacement maps (VDMs) from static magnetic field (B0) maps acquired during each scan session. These VDMs were used to correct for susceptibility-X-movement-related distortions in the EPI images. These distortion-corrected images were then co-registered to the subject's anatomical image. Images were spatially normalized (4th degree B-spline) into a standard stereotactic space (MNI template), re-sampled into 2 mm isotropic voxels, and smoothed with a 6 mm full-width-half-maximum gaussian kernel. We then applied a high-pass filter (128 s cutoff) to remove low-frequency signal drift. Each subject's data were inspected for excessive motion—only subjects with <3 mm motion in every direction across all runs were included in analyses. For single-subject analyses, trials were pooled across the levels of monetary value for a given condition. Onsets for the anticipatory delay period and for the feedback period of each of the three trial types were separately modeled using a canonical hemodynamic response function (HRF) with a time derivative. In addition, six head motion parameter estimates (translation in *x*, *y*, *z*; roll, pitch, yaw) were included as covariates in the design matrix. Each run was modeled separately. We then contrasted the beta-weights of repressors using a *t*-test between trial types to create, for each subject, a contrast image showing voxels that were differentially activated as a function of task conditions.

Based on our *a priori* hypotheses regarding the relationship between perceived stress and corticostriatal function, our group analyses included associations between PSS scores and neural activity during both the anticipatory and feedback phases. For the anticipatory phase, we separately examined the relationship between PSS scores and contrasts of Potential Win Anticipation > No Change Anticipation and Potential Loss Anticipation > No Change Anticipation. Note that these analyses included all trials for each of the conditions regardless of the outcome of the trial. In contrast, analyses of the feedback phase were dependent upon the outcome of the trial. Because we were primarily interested in responses to gains or losses, analysis of the Feedback phase focused on the contrasts of Win Feedback > No Change Feedback and Loss Feedback > No Change Feedback. For the Win Feedback > No Change Feedback contrast, we only modeled trials in which the subject had successfully achieved a “Hit,” meaning they had responded before the target disappeared from the screen, and therefore received feedback indicating a monetary gain of the amount available on the given trial. Potential Win and No Change trials where the subject failed to respond quickly enough (a “Miss”) were not included in this contrast because there was no change in money in those trials. Conversely, for Loss Feedback trials, we only modeled trials in which the subject failed to respond before the target disappeared from the screen (“a Miss”), and received feedback informing them that they had lost money. For the Loss Feedback contrast, we did not model Potential Loss or No Change trials in which the subject achieved a “Hit” and avoided a loss of money because there was no change in money on those trials.

Random effects analyses of fMRI data were performed in SPM5 by regressing subjects' perceived stress scores against contrast images with subject sex and scanner as covariates in the model. While effects of sex on reward processing were not the focus of the current study, past studies have suggested the possibility of sex differences in response to stress (e.g., Mather and Lighthall, 2012). Consequently, to control for the possible differences of sex, we included it as a covariate. This approach has been used in a number of prior publications involving individual differences in reward processing from our lab (e.g., Buckholtz et al., 2010).

All analyses were whole-brain, and SPMs were explored using a voxel-wise threshold of *p* < 0.005 (uncorrected) and a minimum cluster extent of 20 voxels. Whole-brain correction for multiple comparisons was achieved using a cluster-extent correction procedure as implemented in SPM5. Only results surviving this cluster-correction (*p*_cluster_ < 0.05) are reported. For contiguous clusters that spread across multiple regions, the automated labeling atlas (AAL) was used to divide clusters so as to differentiate between structures. After significant clusters had been identified, parameter estimates reflecting task-dependent changes in BOLD signal for each subject were extracted and entered into SPSS19 (IBM, Armonk, NY) for the purposes of visualization.

## Results

### PSS scores

Subject scores on the 10-item PSS ranged from 0 to 32 (*M* = 14.7, *SD* = 7.5) out a maximum possible score of 40 on the instrument. These results are consistent with normative data on this instrument for subjects within this age range (*M* = 14.2, *SD* = 6.2) (Cohen and Williamson, 1988).

### Neuroimaging data: MID results

#### Win and loss feedback

Consistent with numerous prior studies using the MID task, a contrast of Win Feedback > No Change Feedback revealed increased BOLD signal in bilateral mPFC encompassing aspects of pregenual cingulate and medial prefrontal gyrus (Peak: *x* = −6, *y* = 44, *z* = −2; *Z*-score = 6.13; *k* = 763; *p*_cluster_ < 0.001) [all coordinates are given in the stereotactic space of the Montreal Neurological Institute (MNI)]. A similar region of mPFC of was identified in the processing of monetary losses during the contrast of Feedback Loss > No Change Feedback, where subjects received feedback that they had missed the target and therefore experienced a monetary loss (Peak: *x* = −8, *y* = 48, *z* = 14; *Z*-score = 4.01, *k* = 140, *p*_cluster_ = 0.034) (see Table 1).

**Table 1:** **Brain regions activated during reward anticipation and feedback conditions of the MID task**.

**Region**	***x***	***y***	***z***	***Z*-score**	***k***	***p* (cluster)**
**REWARD FEEDBACK:WIN > NO CHANGE**						
Medial prefrontal cortex	−6	44	−2	6.13	763	<0.001
R posterior hippocampus	24	−40	0	4.90	190	0.004
**REWARD FEEDBACK: LOSS > NO CHANGE**						
Medial prefrontal cortex	−8	48	14	4.01	140	0.034
**REWARD ANTICIPATION:WIN > NO CHANGE**						
L ventral striatum	−6	8	−4	7.81	611	<0.001
R ventral striatum	12	14	−4	7.29	647	<0.001
L anterior insula	−28	18	−4	7.29	685	<0.001
R anterior insula	36	20	−8	6.76	467	<0.001
L cerebellum	−32	−54	−22	6.98	3800	<0.001
R cerebellum	8	−66	−10	7.15	3800	<0.001
L thalamus	−8	−14	10	6.91	1068	<0.001
R thalamus	4	−14	8	6.77	1068	<0.001
L amygdala	−20	0	−14	6.73	103	0.048
R amygdala	18	4	−16	6.54	121	0.025
L hippocampus	−16	−26	−10	6.70	269	<0.001
R hippocampus	18	−24	−12	6.34	152	0.004
Medial prefrontal cortex/dorsal ACC	0	30	26	5.72	810	<0.001
**REWARD ANTICIPATION: LOSS > NO CHANGE**						
L anterior insula	−28	18	−4	6.18	505	<0.001
R anterior insula	36	20	−8	8.95	398	<0.001
L cerebellum	−30	−56	−20	7.35	3907	<0.001
R cerebellum	8	−66	−10	7.26	3907	<0.001
L ventral striatum	−8	10	−4	6.47	548	<0.001
R ventral striatum	10	8	4	7.28	628	<0.001
L amygdala	−20	0	−12	6.73	105	0.047
R amygdala	20	2	−14	6.65	125	0.024
L thalamus	−8	−14	10	6.71	1031	<0.001
R thalamus	4	−14	10	6.41	1031	<0.001
L hippocampus	−20	−26	−8	6.26	197	0.001
R hippocampus	18	−28	−8	5.44	89	0.042
Medial prefrontal cortex/dorsal ACC	−2	32	26	5.12	382	<0.001

#### Potential reward and loss anticipation

Also in keeping with prior findings using the MID, we observed robust activation in the ventral striatum during the contrast of Potential Win Anticipation > No Change Anticipation, as well as activity in amygdala, hippocampus, insula, mPFC, thalamus and cerebellum. A similar pattern of activation was obtained during the contrast of Potential Loss Anticipation > No Change Anticipation (see Table 1).

### Neuroimaging data: correlations with perceived stress

#### Reward and loss feedback

**Figure 2:**
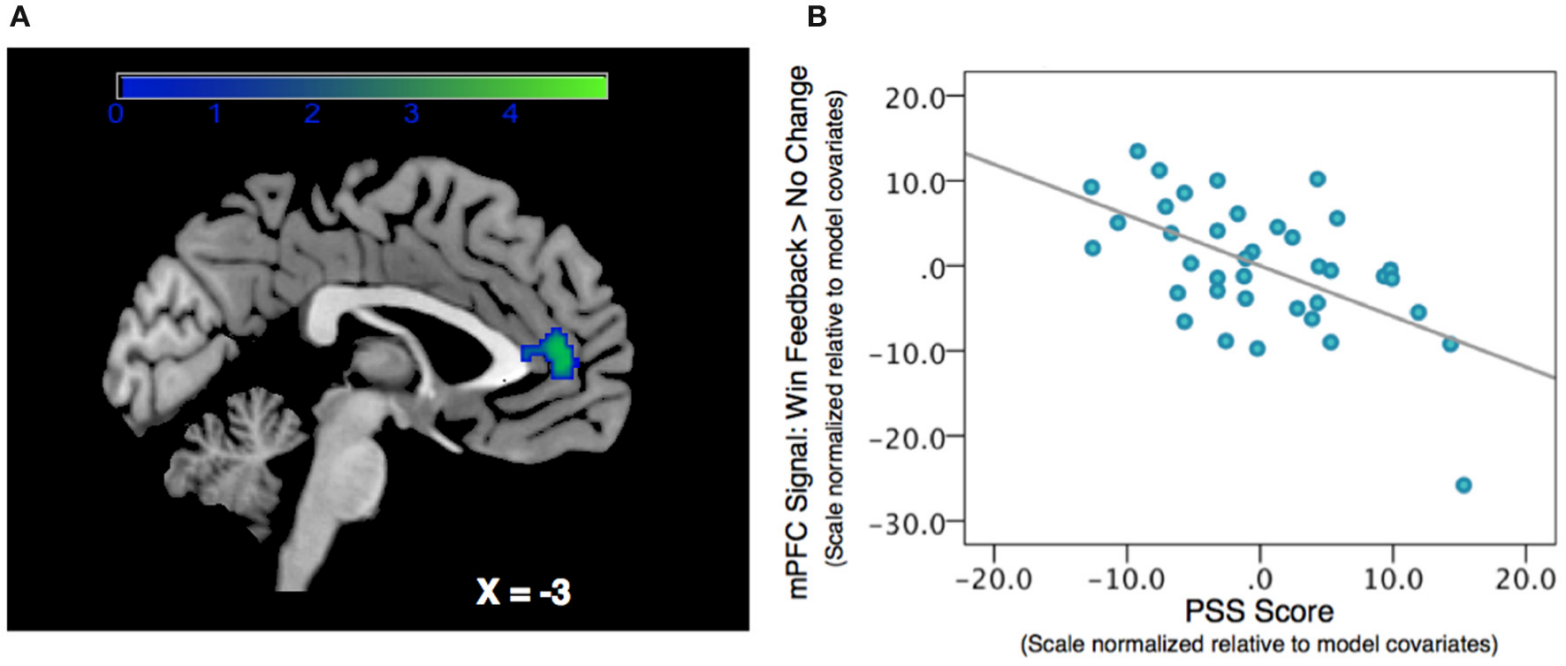
**Association between Perceived Stress and mPFC BOLD signal during a contrast of Win Feedback > No Change Feedback. (A)** SPM depicting mPFC cluster. Cluster is significant after correcting for multiple-comparisons using a cluster-extent correction procedure *p*_cluster_ = 0.023. Color-bar indicates *t*-statistic. **(B)** Partial regression plot, which normalizes variables relative to model-covariates, depicting the relationship between perceived stress and mPFC BOLD response during Win Feedback > No Change Feedback. NB: the effect is still significant when the potentially influential data point in the bottom right corner of the graph is removed.

We regressed PSS scores against reward feedback activity during the Win Feedback > No Change Feedback contrast, and found a significant inverse association in bilateral mPFC, primarily in pregenual ACC and cingulate sulcus (Peak: *x* = 0, *y* = 50, *z* = 4; *Z*-score = 3.53; *k* = 132, *p*_cluster_ = 0.023) (see Table 2; Figure 2). This association suggests that individuals reporting higher levels of stress in the preceding month exhibited diminished amounts of BOLD signal in this region.

**Table 2:** **Brain regions showing an association with PSS scores**.

**Region**	***x***	***y***	***z***	***Z*-score**	***k***	***p* (cluster)**
**REWARD FEEDBACK: WIN > NO CHANGE**						
Medial prefrontal cortex	0	50	4	3.53	132	0.023
**REWARD FEEDBACK: LOSS > NO CHANGE**						
Medial prefrontal cortex	−8	48	14	4.01	140	0.034
L anterior insula	−6	46	8	3.62	132	0.041
**REWARD ANTICIPATION: WIN > NO CHANGE**						
–	–	–	–	–	–	–
**REWARD ANTICIPATION: LOSS > NO CHANGE**						
–	–	–	–	–	–	–

**Figure 3:**
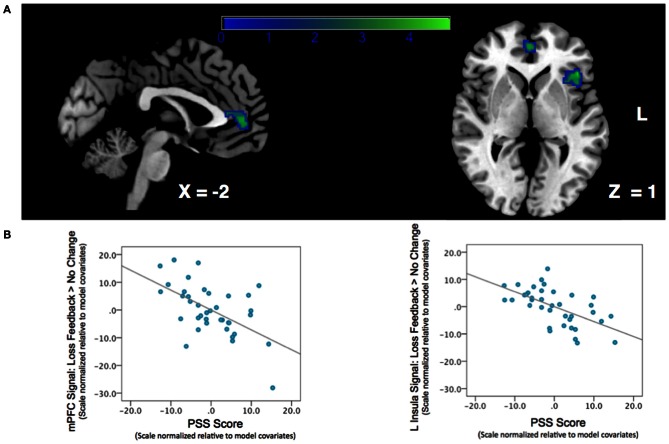
**Association between Perceived Stress and mPFC BOLD signal during a contrast of Loss Feedback > No Change Feedback. (A)** SPM depicting mPFC and insula clusters. Clusters are significant after correcting for multiple-comparisons using a cluster-extent correction procedure *p*_cluster_ < 0.05. Color-bar indicates *t*-statistic. **(B)** Partial regression plots depicting the relationship between perceived stress and BOLD response during Loss Feedback > No Change Feedback in mPFC and left anterior insula. NB: the effect is still significant when potentially influential data point in the bottom right corner of the graph is removed.

We next examined the relationship between perceived stress and reward feedback activation during the Loss Feedback > No Change Feedback contrast, and again found a significant inverse association in mPFC (Peak: *x* = −6, *y* = 46, *z* = 8; *Z*-score = 3.62; *k* = 132; *p*_cluster_ = 0.041) as well as a region of left anterior insula (Peak: *x* = −44, *y* = 26, *Z*-score = 4.17; *k* = 182; *p*_cluster_ = 0.009) (see Table 2; Figure 3). This finding suggests that higher PSS scores were associated with reduced neural responses in both mPFC and insula when subjects received feedback that they had experienced a monetary loss.

#### Potential reward and loss anticipation

There were no suprathreshold voxels showing an association between PSS scores and neural activity during the anticipation phase for either the Potential Win Anticipation > No Change or Potential Loss Anticipation > No Change contrasts.

## Discussion

The present study tested the relationship between individual differences in perceptions of recent life stressors and corticostriatal circuit functioning during reward processing. We found that higher levels of perceived stress were associated with diminished neural responses in the mPFC when subjects received feedback about monetary rewards and losses. These findings support a growing body of evidence implicating the mPFC as a critical region for stress-linked changes in reward processing.

The localization to mPFC is notable for several reasons. First, mPFC is known to be structurally vulnerable to chronic stress. Numerous independent studies in animals have shown that chronic stress incites a retraction of dendritic morphology within the mPFC (Cook and Wellman, 2004; Radley et al., 2005, 2006a,b; Cerqueira et al., 2007); for a review, see McEwen (2007), impairing its capacity to communicate with other striatal and limbic regions involved in reward salience and learning (Dias-Ferreira et al., 2009). While the mechanisms of this susceptibility are not fully understood, strong evidence suggests that prefrontal glucocorticoid elevations play a key role (McEwen, 2007): along with the hippocampus, the mPFC expresses a high number of glucocorticoid receptors (Chao et al., 1989; Ahima and Harlan, 1990; Patel et al., 2000), and participates in negative feedback regulation of glucocorticoid release (Akana et al., 2001; Mizoguchi et al., 2003). Further, site-injections of glucocorticoids have been found to mimic the structural consequences of chronic stress within mPFC (Wellman, 2001; Cerqueira et al., 2005a,b, 2007). Consistent with these preclinical findings, elevated cortisol levels in humans have been found to correlate with reduced gray matter volume in this region (Castro-Fornieles et al., 2009; Treadway et al., 2009).

Such stress-related microdamage in mPFC impacts a variety of cognitive processes (Liston et al., 2006; McEwen, 2007). In the context of reward, stressors can increase habitual response patterns that are insensitive to changing reinforcement context. (Schwabe and Wolf, 2009; Soares et al., 2012). Importantly, this stress-induced shift toward habitual responding has been linked to diminished mPFC activity in response to reward information (Schwabe et al., 2012). Consistent with the current findings, these data suggest that stress-induced shifts in mPFC function—possibly reflecting structural microdamage (Dias-Ferreira et al., 2009)—may impair appropriate encoding of value information. This proposed role for mPFC function is consistent with electrophysiological data recorded in non-human primates, where individual neurons within mPFC—especially the ACC and cingulate sulcus—have been shown to play a vital role in incorporating reward feedback information as a means of encoding action-outcome relationships and updating values for subsequent behaviors (Wallis and Kennerley, 2010). Our data would appear to corroborate this model, suggesting that elevated stress reduces the capacity to accurately encode the appropriate salience of new information. In keeping with this proposal, individual differences in the PSS have been previously linked to decreased sensitivity to reinforcement information during a signal detection task (Pizzagalli et al., 2007).

Somewhat unexpectedly, we did not observe any associations between perceived stress and neural activity during the anticipation phase. On the surface, this is surprising, as several fMRI studies using acute stressors have observed direct effects on reward anticipation and anticipatory decision-making, rather than reward feedback (Ossewaarde et al., 2011; Mather and Lighthall, 2012; Porcelli et al., 2012). This discrepancy may partly reflect the fact that unlike studies that use an acute, in-the-moment stress manipulation to examine neural responses to stress (Ossewaarde et al., 2011; Mather and Lighthall, 2012; Porcelli et al., 2012), the current study used the PSS to test the association between a recent history of elevated stress perceptions to reward and loss processing. It is increasingly recognized that the neural mechanisms governing acute vs. chronic stressors are somewhat distinct (Cabib and Puglisi-Allegra, 2011). Moreover, animal models suggest that it is chronic stress that is most likely to affect the various forms of structural microdamage in mPFC discussed above. Consequently, the selective associations between PSS scores and feedback-related activity may reflect the duration of stress that is captured by the PSS. In addition to this temporal dimension, the PSS assesses subjects' perceptions of their ability to cope with, control and adapt to stressful experiences. Perceived controllability has marked effects on the neurobiological consequences of stress, and has similarly been localized to mPFC (Cabib and Puglisi-Allegra, 1994; Amat et al., 2005, 2008; Pascucci et al., 2007; Maier and Watkins, 2010). Additional research will be required to fully understand the implications of these divergent responses in mPFC as a function of chronicity and subjective perception. That said, it should be emphasized that it is stressors that are experienced as being chronic, unpredictable and uncontrollable that are most likely to increase risk for psychopathology, rather than acute stressors (Docherty, 1996; Kessler, 1997; Kendler et al., 2004; Hammen, 2005).

It is also worth commenting on the similar pattern of results observed for both the “Win” and “Loss” conditions. This stands in contrast with a number of recent papers showing divergent effects of stress on reward learning and decision-making, where acute stress has been found to selectively facilitate learning about wins while impairing learning about punishment (Petzold et al., 2010; Cavanagh et al., 2011; Mather and Lighthall, 2012; Porcelli et al., 2012). Interestingly, one distinction that emerged between the two conditions was that perceived stress was associated with decreased left anterior insula activity during the Loss trials, but not the Win trials. The anterior insula is increasingly recognized as an important region in value-based decision-making in general (Weller et al., 2009; Treadway et al., 2012) and punishment-avoidance learning in particular (Kim et al., 2006; Pessiglione et al., 2006; Samanez-Larkin et al., 2008; Palminteri et al., 2012). Moreover, alterations in anterior insula activity during reward decision-making have been observed as a consequence of stress (Mather and Lighthall, 2012). Given the apparent valence-specific role of the anterior insula in avoidance-learning, it is intriguing that neural activity in this region showed an association with perceived stress only during the loss condition. As with mPFC, reduced activity in this region during feedback may contribute to decreased encoding of reinforcer information following stress.

In sum, the current findings help identify how variation in perceived stress influence neural circuitry involved in responding to reward feedback information. Understanding how the brain is affected by elevated stress load is important for understanding stress-linked risk for psychopathology. Our findings primarily highlight the mPFC, which is widely implicated in a number of fundamental cognitive processes related to affect regulation (Ochsner and Gross, 2005; Etkin et al., 2006), value-based decision-making (Rushworth et al., 2004; Wallis and Kennerley, 2010), and self-evaluation and negative self-judgment (Enzi et al., 2009). Importantly, structural, functional, and neurochemical alterations in mPFC have been reported across a number of psychiatric diagnoses (Coryell et al., 2005; Fitzgerald et al., 2008; Goldstein et al., 2009; Koch et al., 2009; Shin et al., 2009; Fineberg et al., 2010; Treadway and Zald, 2011; Gabbay et al., 2012; Keating et al., 2012). Taken together these findings implicate mPFC as a transdiagnostic nexus, wherein dysfunction predisposes diverse forms of psychopathology that, while categorically distinct, may be symptomatically related due to shared deficits in mPFC-subserved cognitive processes (Buckholtz and Meyer-Lindenberg, 2012). While our study did not include a patient sample, the present data indicate that associations with perceived stress can be observed even in samples with no overt psychopathology. Given the well-known link between perceived stress and the risk for developing such disorders, our data support the hypothesis that the mPFC is a critical node of vulnerability for developing stress-linked reward processing symptoms.

### Conflict of interest statement

The authors declare that the research was conducted in the absence of any commercial or financial relationships that could be construed as a potential conflict of interest.
